# The tumour-associated glycoprotein podoplanin is expressed in fibroblast-like synoviocytes of the hyperplastic synovial lining layer in rheumatoid arthritis

**DOI:** 10.1186/ar3274

**Published:** 2011-03-07

**Authors:** Anna-Karin H Ekwall, Thomas Eisler, Christian Anderberg, Chunsheng Jin, Niclas Karlsson, Mikael Brisslert, Maria I Bokarewa

**Affiliations:** 1 Department of Rheumatology and Inflammation Research, Institute of Medicine, Sahlgrenska Academy, Göteborg University, Box 480, 405 30 Göteborg, Sweden; 2 Department of Orthopaedics, Institute of Clinical Sciences, Sahlgrenska Academy, Göteborg University, Bruna Stråket 11, 314 45 Göteborg, Sweden; 3 Division of Orthopaedics, Spenshult Hospital, 313 92 Oskarström, Sweden; 4 Department of Medical Biochemistry and Cell Biology, Institute of Biomedicine, Sahlgrenska Academy, Göteborg University, Box 440, 405 30 Göteborg, Sweden

## Abstract

**Introduction:**

Activated fibroblast-like synoviocytes (FLSs) in rheumatoid arthritis (RA) share many characteristics with tumour cells and are key mediators of synovial tissue transformation and joint destruction. The glycoprotein podoplanin is upregulated in the invasive front of several human cancers and has been associated with epithelial-mesenchymal transition, increased cell migration and tissue invasion. The aim of this study was to investigate whether podoplanin is expressed in areas of synovial transformation in RA and especially in promigratory RA-FLS.

**Methods:**

Podoplanin expression in human synovial tissue from 18 RA patients and nine osteoarthritis (OA) patients was assessed by immunohistochemistry and confirmed by Western blot analysis. The expression was related to markers of synoviocytes and myofibroblasts detected by using confocal immunofluoresence microscopy. Expression of podoplanin, with or without the addition of proinflammatory cytokines and growth factors, in primary human FLS was evaluated by using flow cytometry.

**Results:**

Podoplanin was highly expressed in cadherin-11-positive cells throughout the synovial lining layer in RA. The expression was most pronounced in areas with lining layer hyperplasia and high matrix metalloproteinase 9 expression, where it coincided with upregulation of α-smooth muscle actin (α-sma). The synovium in OA was predominantly podoplanin-negative. Podoplanin was expressed in 50% of cultured primary FLSs, and the expression was increased by interleukin 1β, tumour necrosis factor α and transforming growth factor β receptor 1.

**Conclusions:**

Here we show that podoplanin is highly expressed in FLSs of the invading synovial tissue in RA. The concomitant upregulation of α-sma and podoplanin in a subpopulation of FLSs indicates a myofibroblast phenotype. Proinflammatory mediators increased the podoplanin expression in cultured RA-FLS. We conclude that podoplanin might be involved in the synovial tissue transformation and increased migratory potential of activated FLSs in RA.

## Introduction

Rheumatoid arthritis (RA) is a chronic systemic inflammatory disease predominantly affecting joints, leading to tissue destruction and functional disability [1,2]. Both genetic and environmental factors are believed to contribute to the dysregulated immune responses seen in this heterogeneous autoimmune disease [3]. Today, treatment strategies involve traditional disease-modifying antirheumatic drugs as well as biologic agents targeting proinflammatory cytokines (tumour necrosis factor α (TNFα), interleukin (IL)-1 and IL-6), B cells or the activation of T cells [4]. Despite this arsenal of drugs, at least 30% of the patients are resistant to the available therapies, suggesting that yet other mediators must be important.

The most prominent feature of RA is the progressive destruction of articular cartilage and bone, which is orchestrated by activated RA fibroblast-like synoviocytes (RA-FLSs) [5,6]. RA-FLSs not only mediate tissue destruction but also are considered to play a major role in initiating and driving RA in concert with inflammatory cells [7]. In the healthy synovium, one to three layers of synoviocytes, the macrophage-like type A and the more abundant fibroblast-like type B (also referred to as synovial fibroblast), form the synovial lining layer separating the synovial sublining layer of loose connective tissue from the joint cavity [8,9]. The synoviocytes are interconnected with adherens junctions containing cadherin-11 [10,11] and E-cadherin [12,13] and are embedded in a lattice of extracellular matrix (ECM) resembling an epithelium but lacking a discrete basal membrane as well as gap junctions and desmosomes. Apart from being a marker of FLSs, cadherin-11 has been shown to be essential for the formation of synovial lining structures *in vitro *and for the development of inflammatory arthritis in mice [14,15].

The morphological hallmarks of RA include activation of FLSs; infiltration of inflammatory cells such as T cells, B cells and macrophages in the sublining; hyperplasia of the synovial lining layer; fibrotic deposition; and subsequent formation of the "pannus" [16]. This tissue mass expands and attaches to and invades the adjacent cartilage and subchondral bone [17]. The major cell type accounting for the thickened lining layer as well as for pannus formation is believed to be activated FLSs [18,19]. These aggressive cells share many characteristics with tumour cells, with upregulated expression of proto-oncogenes and promigratory adhesion molecules, increased production of proinflammatory cytokines and matrix-degrading enzymes [7], as well as increased resistance to apoptosis [20,21]. There are data indicating that the transformed phenotype of RA-FLS is stable and maintained even in the absence of stimulus from inflammatory cells [22]. In high-inflammation synovial tissue, RA-FLSs show a gene expression profile characteristic of myofibroblasts, and cells of the synovial lining in RA have been found to express α-smooth muscle actin (α-sma) and type IV collagen [13,23]. Thus, it has been suggested that RA-FLSs can undergo a process resembling epithelial-mesenchymal transition (EMT), a phenomenon known from early developmental processes, tissue repair, fibrosis and carcinogenesis [24,25]. Recently, it was also suggested that migrating RA-FLSs might be responsible for spreading the disease to distant joints [26].

Podoplanin (identical to human PA2.26, aggrus and T1α-2), is a small, 38- to 40-kDa, mucin-type transmembrane glycoprotein normally expressed on human lymphatic endothelia, basal epithelial keratinocytes, myoepithelial cells and myofibroblasts of certain glandular tissues, follicular dendritic cells and fibroblastic reticular cells of lymphoid organs and alveolar type I cells [27,28]. We demonstrated strong podoplanin expression on subepithelial interstitial cells in human endolymphatic tissue of the inner ear [29]. The physiologic function of podoplanin is to a large extent unknown, but knockout (KO) studies showed that it is crucial for the development of the lung and deep lymphatics in mice [28]. The podoplanin-KO mice died at birth as a result of respiratory failure and generalised lymphoedema. Overexpression of this glycoprotein in epithelial cells induced a dentritic cell morphology and increased cell adhesion and migration [27]. Interestingly, increasing data show that podoplanin is upregulated on the invasive front of human cancers [27,30]. The expression of podoplanin is correlated with metastasis and a bad prognosis. In addition, podoplanin (or aggrus) induces platelet aggregation of tumour cells [31] and has been associated with both EMT-dependent and EMT-independent tumour cell invasion [32]. There are a few studies indicating increased podoplanin expression in fibroblasts in reactive tissues, such as in chronic pleuritis, in cancer-associated fibroblasts [33] and in cultured fibroblasts [34]. However, little is known about the potential role of podoplanin in inflammation and tissue repair. In this study, we were interested to see whether podoplanin is expressed in FLSs in RA and could be associated with the fibrotic transformation of the synovium in this disease.

## Materials and methods

### Human synovial tissue and cells

Synovial tissue specimens and fluid were obtained from patients with RA (*n *= 18) or OA (*n *= 9) during joint replacement surgery or therapeutic joint aspiration at Sahlgrenska University Hospital and Spenshult Hospital in Sweden. Both weight-bearing (knee and hip) and non-weight-bearing (shoulder and elbow) joint specimens were included. All RA patients fulfilled the American College of Rheumatology 1987 revised criteria for RA [35]. Preoperative radiographs were scored according to Larsen index (1 to 5) [36]: 0 = normal; 1 = slight abnormality, soft tissue swelling, periarticular osteoporosis and slight joint space narrowing; 2 = early abnormality, erosions (obligatory in non-weight-bearing joints) and joint space narrowing; 3 = medium destructive abnormality, erosions and joint space narrowing; 4 = severe destructive abnormality, erosions, joint space narrowing and bone deformation; and 5 = mutilating abnormality. The patient characteristics are outlined in Table 1. All patients gave informed consent, and the procedure was approved by the Ethics Committee of Gothenburg in Sweden. Human primary FLS cultures were established as follows: representative tissue pieces were minced, treated with 1 mg/ml collagenase/dispase (Roche, Mannheim, Germany) for 1 hour at 37°C and passaged through a cell strainer. The cell suspension was rinsed twice in phosphate-buffered saline (PBS), resuspended in Dulbecco's modified Eagle's medium (DMEM) GlutaMAX (Invitrogen, Camarillo, CA, USA) supplemented with 10% heat-inactivated foetal bovine serum (HIFBS) (Sigma, St. Louis, MO, USA), 50 μg/ml gentamicin (Sanofi-Aventis, Paris, France) and 100 μg/ml normocin (Invivogen, San Diego, CA, USA) and incubated at 5% CO_2 _at 37°C. Cells in passages 3 through 6 were used.

**Table 1:** Characteristics of patients^a^

Characteristic	RA (*n *= 18)	OA (*n *= 9)
Age, mean yr	61.8	68.4
Sex, F/M	13/6	6/3
Disease duration, mean yr	21.9	-
Seropositive^b^, %	82%	-
Larsen score^c ^(mean ± SD)	2.9 ± 0.6	-
DMARDs, %	72%	-
Steroids, %	44%	-
Biologic drugs, %	33%	-

^a^RA, rheumatoid arthritis; OA, osteoarthritis; DMARDs, disease-modifying antirheumatic drugs; ^b^rheumatoid factor or anticyclic citrullinated peptide antibody-positive; ^c^Larsen index score (1 to 5) of the biopsied joint (bone erosion present if index >1).

### Immunohistochemistry

Paraformaldehyde (PFA)-fixed (Histolab, Göteborg, Sweden), paraffin-embedded (4 μm) or acetone-fixed (Histolab) frozen sections (6 μm) were rehydrated in Tris-buffered saline for 10 minutes. Antigen retrieval was performed when required in a pressure chamber (2100 Retriever; Histolab). Unspecific binding was blocked using serum-free protein block or normal rabbit serum (Dako, Glostrup, Denmark). After incubation with mouse monoclonal antihuman podoplanin (clone D2-40; AbD Serotec, Oxford, UK), mouse monoclonal antihuman cadherin-11 (clone 5B2H5; Invitrogen) or mouse monoclonal antihuman CD90 antibodies (clone AS02; Dianova, Hamburg, Germany), respectively, the specimens were incubated with a biotinylated rabbit antimouse immunoglobulin G F(ab')_2 _fragment (Dako) followed by streptavidin-conjugated alkaline phosphatase (Dako). Fast Red Naphthol (Sigma) was used as a substrate, and the specimens were counterstained with Mayer's haematoxylin (Histolab) and mounted in Aqua-Mount mounting medium (VWR International Ltd, Leicestershire, UK). The same staining protocol was used for immunocytochemistry of primary FLS seeded onto chamber slides (Lab-Tek; Nunc, Rochester, NY, USA) and fixed in PFA. Normal mouse IgG1 (Dako) was used as a negative control. The podoplanin staining was scored by two independent observers blinded to the procedure according to the following scoring method: 0 = negative staining, 1 = positive staining of single or limited groups of cells in the lining layer, 2 = continuous positive staining of the cells of the synovial lining layer and 3 = same as 2, but with the addition of positive staining of cells in the sublining layer.

### Immunofluorescence and confocal microscopy

Paraffin-embedded synovial sections were subjected to a double-staining procedure: incubation with rabbit antihuman cadherin-11 (Invitrogen), rabbit anti-matrix metalloproteinase (MMP)-9 (AB805; Millipore, Billerica, MA, USA), rabbit antihuman E-cadherin (clone H-108; Santa Cruz Biotechnology, Santa Cruz, CA, USA) or rabbit anti-α-sma (PA1-37024; Thermo Scientific, Rockford, IL, USA) antibodies followed by addition of Alexa Fluor 555-conjugated goat antirabbit IgG (Invitrogen) or, in one step, Alexa Fluor 647-conjugated mouse antihuman CD68 (clone KP1; Santa Cruz Biotechnology). Second, mouse antihuman podoplanin (clone D2-40) incubation was followed by Alexa Fluor 488-conjugated goat antimouse IgG (Invitrogen). Alternatively, biotinylated mouse antihuman podoplanin (Acris Antibodies GmbH, Herford, Germany) and Alexa Fluor 488-conjugated streptavidin were added prior to mouse antihuman cadherin-11 (clone 5B2H5) and Alexa Fluor 555-conjugated goat antimouse IgG (Invitrogen). Slides were placed in ProLong Gold antifade reagent mounting medium with 4',6-diamidino-2-phenylindole (Invitrogen). Normal mouse IgG1 or normal rabbit serum (Dako) was used as negative controls. Images were collected using a confocal microscope (LSM700; Zeiss, Oberkochen, Germany). The background fluorescence level was set with the negative controls, and images were analysed using Zen image analysis software 2009 (Zeiss).

### Western blot analysis

Membrane proteins from tissue and cell pellets were prepared by sodium carbonate treatment [37]. In brief, lyophilized material was resuspended in 0.1 M sodium carbonate before sonication. After removal of cell debris, the membrane fraction was collected by ultracentrifugation at 115,000 *g *for 75 minutes. The membrane proteins were solubilised with 7 M urea, 2 M thiourea, 40 mM Tris, 1% C7 detergent (wt/vol) and 4% 3-[(3-cholamidopropyl)dimethylammonio]-1-propanesulfonate buffer (wt/vol) and kept at -80°C before use.

Samples, together with recombinant unglycosylated human podoplanin core protein (ProSpec, Ness-Ziona, Israel), were separated by 20% sodium dodecyl sulphate polyacrylamide gel electrophoresis (SDS-PAGE) under reducing conditions with 10 mM dithiothreitol. After being transferred onto polyvinylidene fluoride membrane, the blots were probed with mouse antihuman podoplanin (1:50; D2-40) and detected with a horseradish peroxidise-conjugated rabbit antimouse antibody (1:2,000; DakoCytomation) and chemiluminescence (SuperSignal West Femto Maximum Sensitivity Substrate; Thermo Scientific).

### Flow cytometry

Primary synovial cell cultures from patients with RA (*n *= 6) and patients with OA (*n *= 5) were trypsinised, resuspended in fluorescence-activated cell sorting buffer (5% HIFBS, 0.09% sodium azide and 0.5% ethylenediaminetetraacetic acid in PBS) and transferred onto a 96-well plate. For intracellular staining (CD68; α-sma), cells were PFA-fixed and permeabilised with 0.1% Triton X-100 in PBS. Unspecific binding was blocked using 1% HIFBS in PBS or Beriglobin P (human IgG; Apoteket, Sweden). Staining was performed with allophycocyanin (APC)-conjugated mouse antihuman CD90, phycoerythrin (PE)-conjugated mouse antihuman CD68, PE-conjugated mouse antihuman CD29 (BD Biosciences, San Jose, CA, USA), mouse antihuman podoplanin (clone D2-40), mouse antihuman cadherin-11 (clone 5B2H5), rabbit antihuman α-sma (PA1-37024) and isotype controls (BD Biosciences). The unconjugated antibodies were incubated with secondary PE-conjugated rat antimouse IgG1 (BD Biosciences) or APC-conjugated goat antirabbit IgG (Santa Cruz Biotechnology) in a second step. Fluorescence was measured using the FACSCanto II system (BD Biosciences) equipped with DIVA 6.2 software (BD Biosciences), and data were analyzed using FlowJo 8.7.3 software (Tree Star Inc., Ashland, OR, USA). The isotype controls were used to set the gates for positive and negative populations.

### Stimulation experiments

Primary FLSs from one OA patient were seeded into complete DMEM in triplicates in six-well plates (100,000 cells/well) and incubated until confluence. The cells were serum-starved in DMEM supplemented with 2% heat-inactivated foetal calf serum for 6 hours before the different human recombinant cytokines were added: 10 ng/ml TNFα (Sigma), 1 ng/ml IL-1β and 1 ng/ml TGF-β1 (R&D Systems, Minneapolis, MN, USA). The cells were harvested by trypsinisation after 12, 24 and 48 hours, and podoplanin expression was measured using flow cytometry with antipodoplanin antibody (clone D2-40). The experiment was repeated four times with different primary cell cultures, including RA-FLSs, with similar results.

### Statistical analysis

Differences in protein expression between the patient groups detected by immunohistochemistry (IHC) and flow cytometry were evaluated using the Mann-Whitney nonparametric test.

## Results

### Podoplanin is expressed in the human synovial lining layer in RA

**Figure 1:**
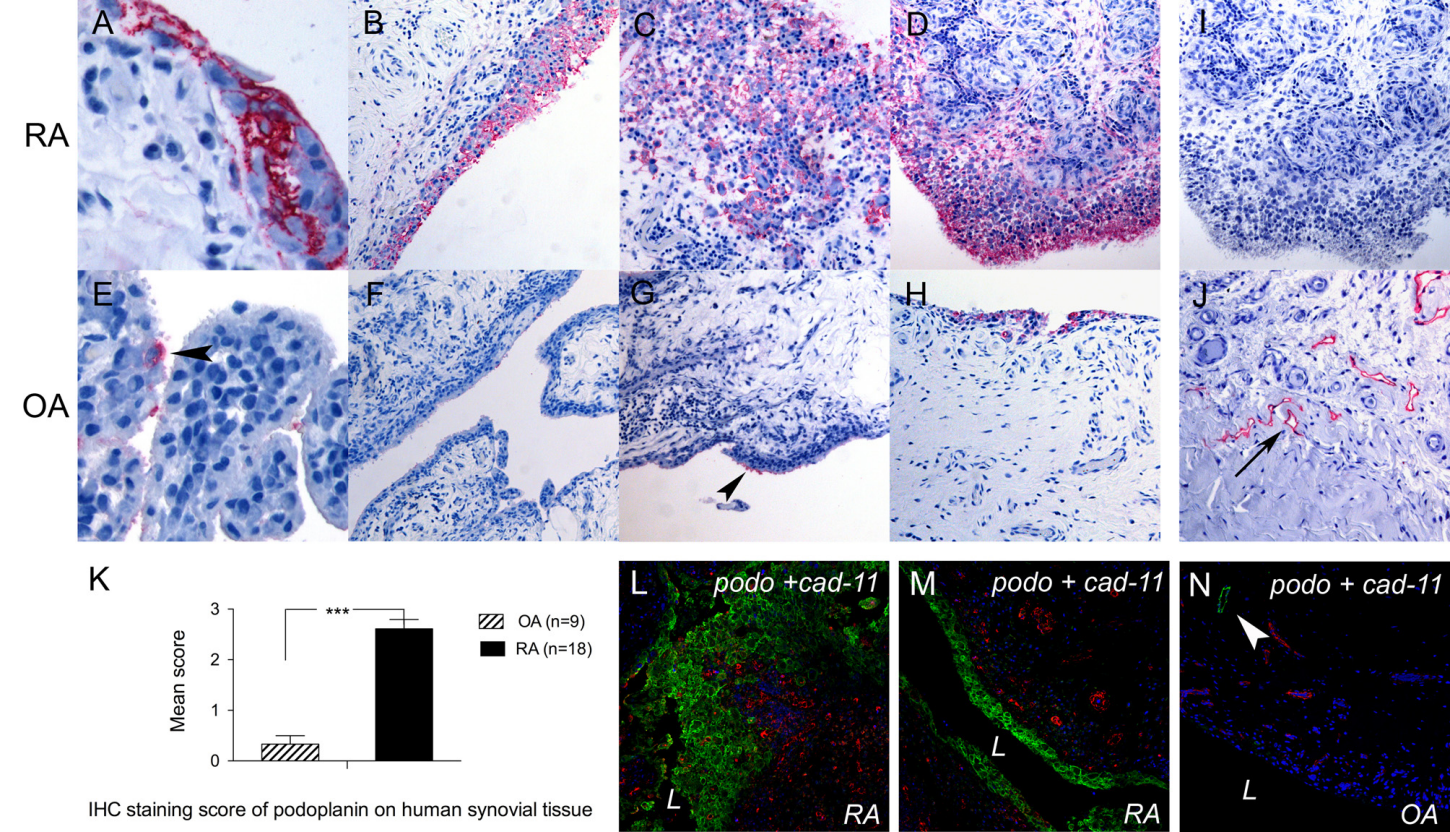
**Podoplanin is expressed in human synovial tissue in RA**. Immunohistochemistry (IHC) of human synovial tissue from **(A-D, I)** rheumatoid arthritis (RA), **(E-H, J) **osteoarthritis (OA) **(A-D, E-H, J) **using antipodoplanin antibody (D2-40) or **(I) **mouse immunoglobulin G_1_. **(J) **Positive control showing lymph vessels (arrow). **(K) **IHC staining score of podoplanin on human synovial tissue from 18 RA and 9 OA patients. Double immunofluorescence staining of **(L and M) **RA and **(N) **OA synovium using antipodoplanin (green) and anti-cadherin-11 (red) antibodies. Note the extensive hyperplasia of the podoplanin-positive lining layer cells **(C and D, L) **and the podoplanin-positive lymph vessel (arrowhead in **N) **but negative lining layer in OA **(N)**. L, lumen. ***statistical significance *P *< 0,0001.

By carrying out IHC on paraffin sections of human synovia, we found that podoplanin was highly expressed in rounded cells of the epithelium-like synovial lining layer in 17 of the 18 RA specimens (Figures 1A-D and 1L-M). In most cases, the podoplanin staining covered the whole cell surface and was continuous along and throughout the lining layer. Podoplanin expression was most pronounced in areas with strong hyperplasia and disrupted synovial architecture (Figures 1C, 1D and 1L), staining not only the surface of all the lining layer cells with high intensity but also adjacent interstitial cells of the sublining layer (Figure 1D). The podoplanin expression was prominent in long cytoplasmatic processes and was maintained on rounded, dispersed and disaggregated cells in "invasive" areas (Figure 1C). Podoplanin stained lymph vessels in all tissues (Figure 1J). The synovium in OA was predominantly negative (Figures 1F and 1N), but single positive cells or a limited group of them were occasionally found in the lining layer (Figures 1E and 1H). Discrete staining was sometimes detected on the apical surface of the outermost lining layer (Figure 1G, arrowhead). The mean score of podoplanin expression in the synovium of the RA specimens was 2.61 (SEM, 0.18) versus 0.33 (SEM, 0.17) for OA specimens (*P *< 0.0001) (Figure 1K). The subsynovial connective tissue in OA was negative in all cases.

**Figure 2:**
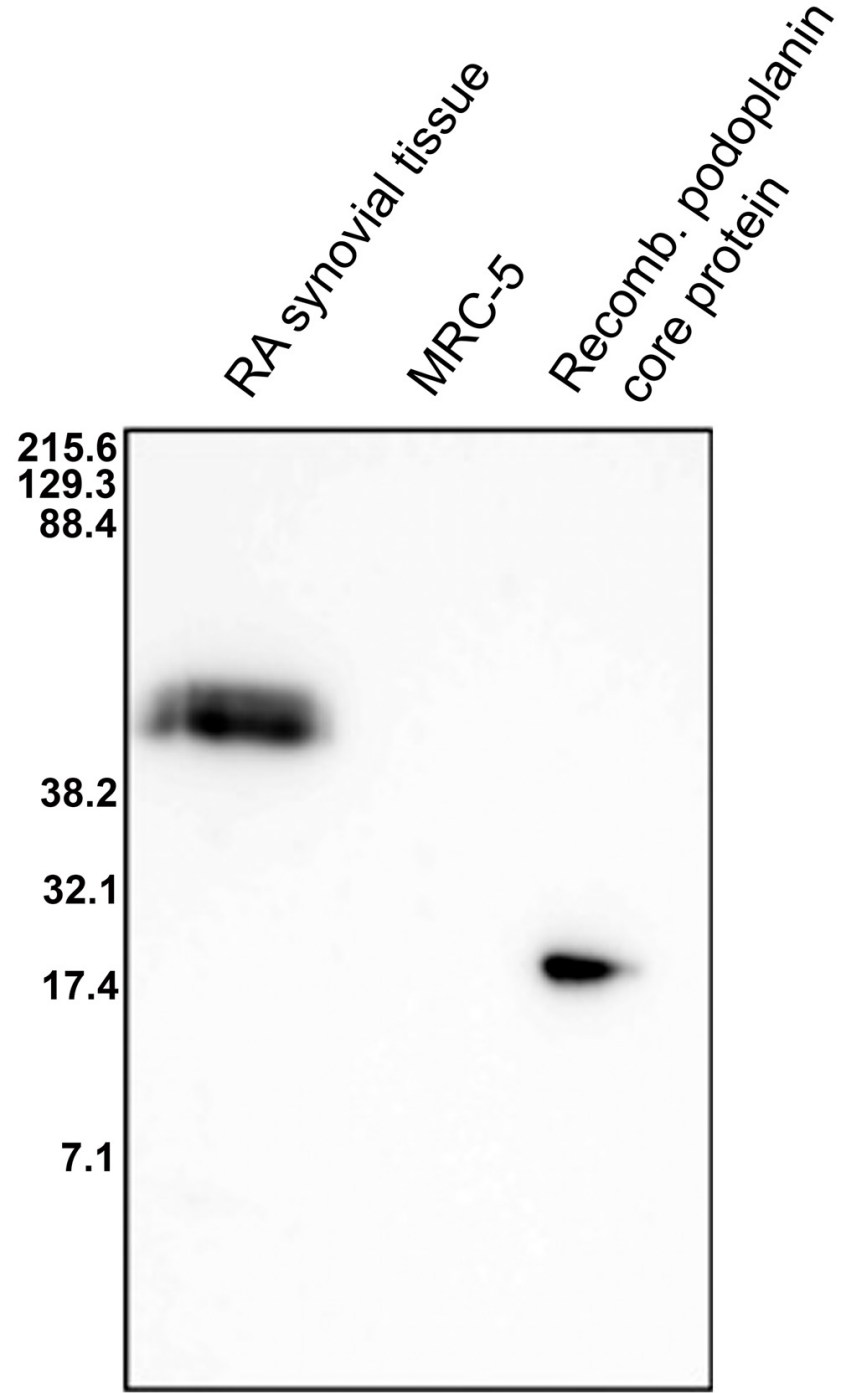
**Anti-podoplanin antibody D2-40 recognizes 45kD band in Western blot of synovial protein extracts**. Western blot of extracted membrane proteins from human synovial tissue from a patient with rheumatoid arthritis (RA) (lane 1), cell pellet of human MRC-5 lung fibroblast cell line (lane 2) and recombinant immature podoplanin core protein (lane 3) separated on a 20% sodium dodecyl sulphate polyacrylamide gel electrophoresis gel probed with the antipodoplanin antibody (D2-40).

To verify that podoplanin is expressed in human synovial tissue in RA and to evaluate the specificity of the antipodoplanin antibody, extracted membrane proteins from synovial tissue samples from two RA patients were subjected to SDS-PAGE and Western blot analysis using D2-40 monoclonal antibody. The Western blot analysis showed one distinct band of about 45 kDa (Figure 2) in both samples. The antibody also recognised the recombinant immature podoplanin core protein (13.4 kDa according to the manufacturer) as a band of estimated molecular weight of about 18 kDa. The lung fibroblast cell line MRC-5, shown by us not to express podoplanin by flow cytometry, was used as a negative control.

### Podoplanin is expressed on cadherin-11-positive synoviocytes of the lining layer in RA

**Figure 3:**
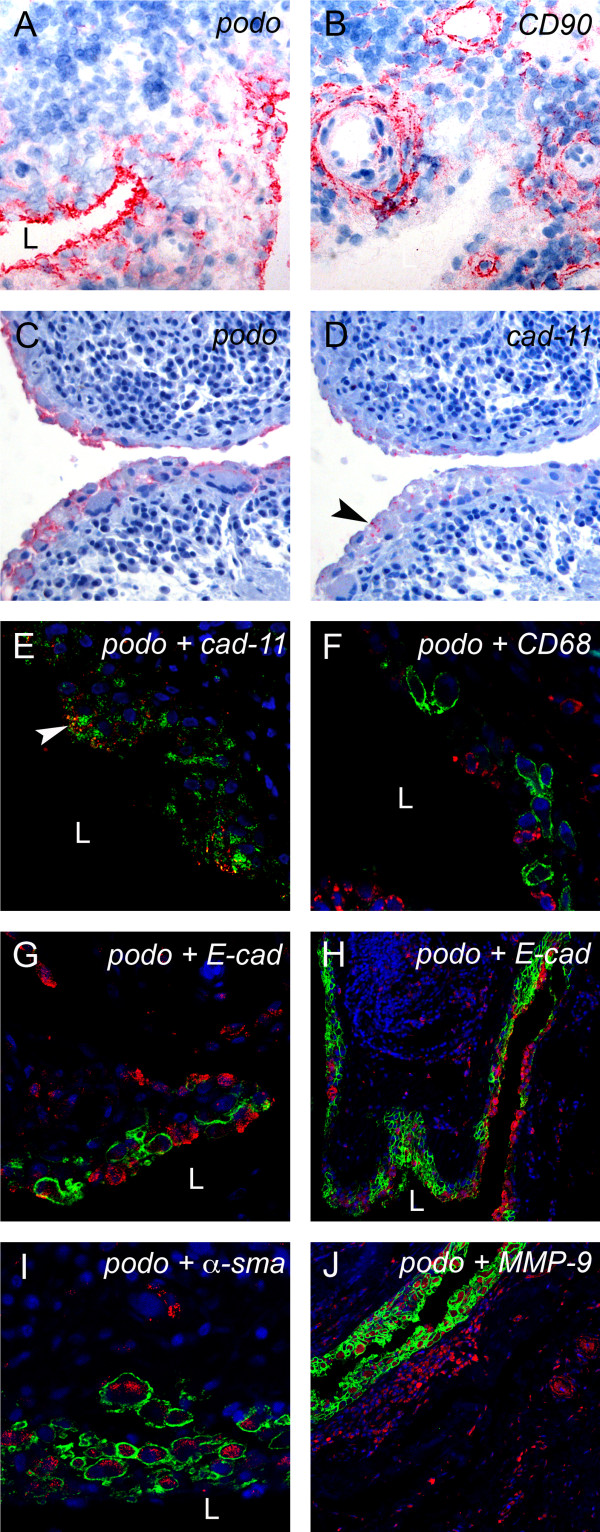
**Podoplanin is expressed in FLS in areas of synovial transformation**. Immunohistochemistry of **(A and B) **frozen and **(C-J) **paraffin-embedded human synovial tissue from patients with rheumatoid arthritis (RA) using antibodies against **(A and C) **podoplanin (D2-40), **(B) **CD90 (AS02) and **(D) **cadherin-11 (3B2H5) (arrowhead). Double immunofluorescence staining analysed by confocal microscopy showing, in green, **(E) **podoplanin 18H5 and **(F-J) **podoplanin D2-40, and in red, **(E) **cadherin-11 (3B2H5), **(F) **CD68 (KP1), **(G **and overview in **H) **E-cadherin (H-108), **(I) **α-smooth muscle actin (α-sma) (PA1-37024) and **(J) **matrix metalloproteinase 9 (MMP-9). **(E) **Note colocalization of podoplanin and cadherin-11 (arrowhead). L, lumen.

To identify which type of synoviocyte express podoplanin, we performed IHC and double-immunofluorescence (double-IF) on human RA synovium using different cellular markers. We found that the fibroblast marker CD90 was expressed by interstitial cells, typically forming sheet structures around capillaries, of the synovial sublining in frozen sections of human synovium (Figure 3B). However, the lining layer was CD90-negative (in contrast to podoplanin) (Figure 3A). Both podoplanin and anti-cadherin-11 were present in the lining layer of serial sections of RA synovia (Figure 3C and 3D). Double-IF staining and confocal microscopy confirmed a colocalization of cadherin-11 and podoplanin on the cellular level of lining cells (Figure 3E). The cadherin-11 expression in RA, compared with OA, was increased both in the lining and in the sublining layers, especially in areas with hyperplasia (Figure 1L). Double-staining for podoplanin and the macrophage marker CD68 clearly did not show any colocalization (Figure 3F). CD68-positive cells were dispersed in the lining layer and in the sublining tissue in both RA and OA.

### α-sma is upregulated in podoplanin-expressing synovial lining layer cells

Next, we were interested to see whether podoplanin could be involved in EMT-like transdifferentiation of RA-FLSs. We therefore investigated the expression of α-sma and E-cadherin in relation to podoplanin in RA synovia. We found that α-sma was expressed in the cytoplasm of podoplanin-positive synovial lining cells in hyperplastic areas (Figure 3I). In addition, α-sma was expressed in vessel walls and on a few dispersed cells in the sublining. E-cadherin could be detected in some areas of the synovial lining layer in both RA and OA specimens. Interestingly, the expression of E-cadherin was very low or absent in podoplanin-expressing lining layer cells (Figure 3G and overview in Figure 3H).

Different MMPs (especially MMP-1, MMP-3, MMP-9 and MMP-13) are upregulated in the RA synovium and are responsible for the degradation of ECM and cartilage [5,38]. We used MMP-9 as an indicator of inflamed synovium and of the presence of matrix degradation. MMP-9 is reportedly expressed in synovial lining cells, in leukocytes and in endothelia of the RA synovium [39]. In agreement with this, we found high expression of MMP-9 in the synovial lining, in sublining ectopic lymphoid structures and in vessels in RA synovial tissue (Figure 3J). Double-staining of podoplanin and MMP-9 showed that the podoplanin-positive lining layer cells expressed MMP-9 (Figure 3J).

### Podoplanin is expressed in cultured CD90-positive FLSs

**Figure 4:**
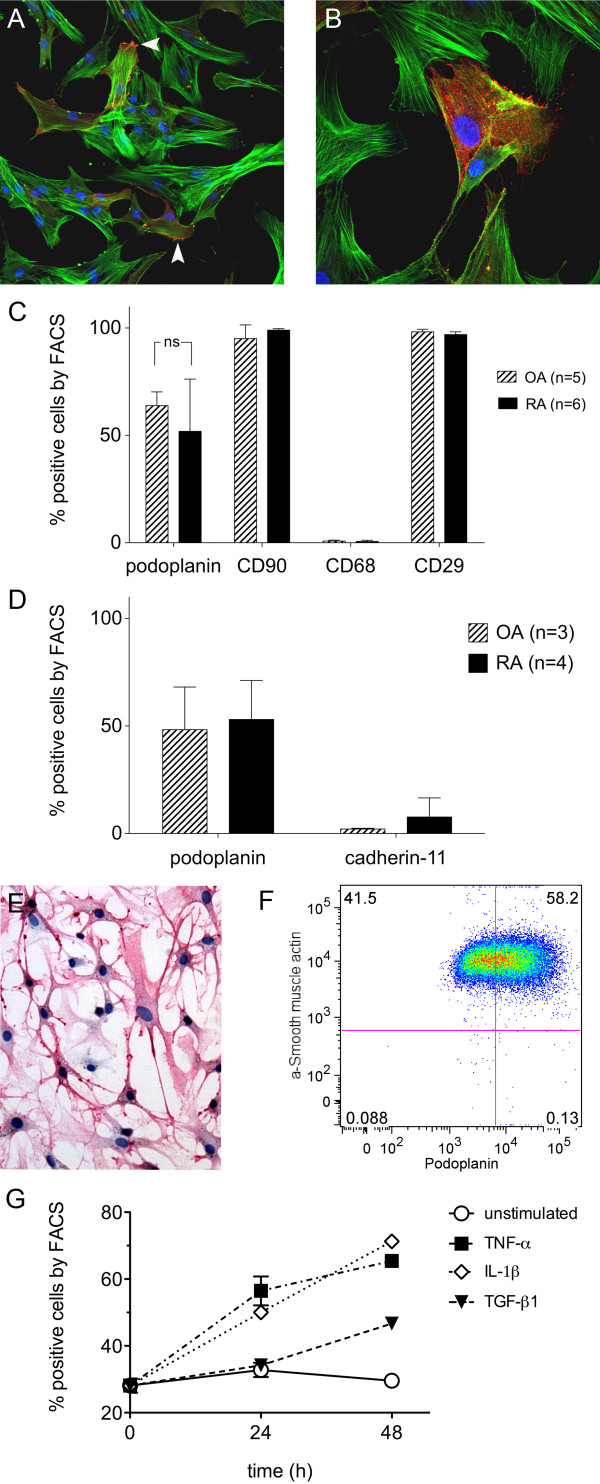
**Podoplanin is expressed in cultured primary FLS and the expression is increased by pro-inflammatory cytokines**. **(A) **Immunofluorescence staining of primary rheumatoid arthritis fibroblast-like synoviocytes (RA-FLS) showing podoplanin (red) and actin stress fibres (green). Note accumulated podoplanin staining in membrane protrusions (arrowheads). **(B) **Magnification of podoplanin-positive RA-FLS. **(C and D) **Flow cytometry (FACS) of primary FLS cultures from patients with RA (filled bars) and patients with osteoarthritis (OA) (striped bars) showing the percentage of positive cells of viable cell populations using **(C) **antipodoplanin and phenotype markers (CD90, CD68 and CD29) and **(D) **cadherin-11 antibodies. **(E) **Immunocytochemistry of an aggressively growing RA-FLS culture using antipodoplanin antibody. Note the dendritic phenotype with long cytoplasmatic protrusions. **(F) **Representative flow cytometry plot of primary RA-FLS stained for podoplanin and α-smooth muscle actin (α-sma) showing the double-positive population (podoplanin^+ ^and α-sma^+^) of 58.2% in the upper right quadrant. **(G) **Graph showing the percentage of podoplanin-positive primary FLS by flow cytometry at baseline, 24 and 48 hours of stimulation with control (complete medium) (open circles), 10 ng/ml tumour necrosis factor (TNF)-α (filled squares), 1 ng/ml interleukin (IL)-1β (open diamond) and 1 ng/ml transforming growth factor β receptor 1 (TGF-β1) (filled triangles), respectively, of a representative culture. The experiment was run in triplicate and repeated four times using different OA-FLS and RA-FLS cultures, which showed similar results but starting at different baseline levels of podoplanin expression.

To characterise podoplanin expression on the cellular level, we established primary FLS cultures from both RA (RA-FLSs) and OA (OA-FLSs) synovial specimens. At passage 3, the cultures were homogeneous. Using IF and confocal microscopy, we found that the primary FLSs had a typical cultured fibroblast phenotype with prominent stress fibres and that approximately 50% of the FLS cells expressed podoplanin (Figure 4A and 4B). The podoplanin expression was most pronounced in areas of focal attachment and in small membrane protrusions (microspikes) (Figure 4A, arrowheads).

The podoplanin expression was further evaluated in six RA-FLS and five OA-FLS primary cell cultures using flow cytometry, showing an average expression of 52 ± 24% and 64 ± 6%, respectively. The variation in RA-FLS compared to OA-FLS was evident. Furthermore, 99 ± 6% of RA-FLSs expressed the fibroblast marker CD90, 97 ± 1% of RA-FLSs expressed CD29 (β1-integrin) and less than 0.6 ± 0.5% of RA-FLSs expressed the macrophage marker CD68. A percentage of 95 ± 0.7% OA-FLS expressed CD90, 98 ± 1% expressed CD29 and less than 0.8 ± 0.4% expressed CD68 (Figure 4C). The mean expression of cadherin-11 was low in OA-FLSs (2.3%) and slightly increased in RA-FLSs (up to 21%) (Figure 4D). Interestingly, one RA-FLS cell line had a more dendritic phenotype (Figure 4E) and was growing without contact inhibition. This cell line had a 100% expression of podoplanin on the basis of flow cytometry.

Cultured fibroblasts upregulate α-sma (up to 100% by passage 5) in culture [40,41]. We were interested to see whether this is true also for primary FLSs. Using flow cytometry, we found that nearly 100% of both OA-FLSs and RA-FLSs express α-sma by passage 6. About 60% (58.2% for RA-FLSs and 61.7% for OA-FLSs) were double-positive for podoplanin and α-sma. All podoplanin-positive cells expressed α-sma (Figure 4F).

### Podoplanin expression *in vitro *is increased by proinflammatory cytokines

IL-1β and TNFα are known to activate RA-FLSs. TGF-β1 is a key mediator of EMT and promotes the differentiation of fibroblasts into myofibroblasts in wound healing and fibrosis.

In this study, we investigated the effects of IL-1β, TNFα and TGF-β1 stimulation on podoplanin expression in primary FLSs. We found a more than twofold increase in podoplanin expression in OA-FLS culture after stimulation with 1 ng/ml IL-1β and 10 ng/ml TNFα. This culture had a low baseline expression of podoplanin (30%), which increased to 72% after 48 hours of IL-1β stimulation (Figure 4G). Using two different primary RA-FLS cultures, we found an early increase in podoplanin expression from, on average, 50% at baseline to about 90% after 12 hours of IL-1β stimulation. These effects were maintained after 36 hours of stimulation (data not shown). TGF-β1 (1 ng/ml) had a moderate effect (+1.7-fold) on podoplanin expression evident at late time points (48 hours) (Figure 4G).

## Discussion

In this study, we have shown that the tumour-associated proinvasive glycoprotein podoplanin is highly expressed in synovial lining layer cells in RA but is rarely found in OA synovial specimens. The expression of podoplanin was most pronounced in areas with signs of inflammation (that is, the presence of leukocyte infiltrates and ectopic lymphoid structures) and synovial transformation (indicated by lining layer hyperplasia, MMP-9 expression and upregulation of cadherin-11 and α-sma). Furthermore, the podoplanin-expressing lining layer cells expressed cadherin-11 but not the macrophage marker CD68, suggesting that these synoviocytes were FLSs rather than synovial macrophages.

All included RA patients had progressed to erosive disease (Larsen index score >1), and all except one had a high podoplanin expression score (IHC score >1) of the synovial tissue from the replaced joint (Table 1). However, without the rarely available tissue specimens from nonerosive and early RA joints to compare these tissues with, we could not analyze whether there is a correlation between erosive disease and podoplanin expression.

The function of podoplanin is far from elucidated. On one hand, this small glycoprotein is constitutively expressed on the apical surface of lymph endothelia as well as on specialised epithelia (for example, podocytes) facing fluid compartments [28,42,43]. On the other hand, podoplanin is crucial for processes involving cell migration, such as the specific embryologic development of deep lymphatics [28] and the invasion and metastasis of certain tumour cells or tissues [32]. Podoplanin has been shown to bind ezrin, an actin filament membrane linker protein, on the inside of the cell *in vitro *[30,44]. It has therefore been suggested that podoplanin is involved in directing actin polymerisation, thereby forming the cellular protrusions needed for migration.

In our study, the marked and widespread expression of podoplanin in lining layer cells in RA was not restricted to the apical cell surface. Instead, it resembled the strong whole cell surface-staining pattern of podoplanin in tumour tissues [30]. It has been shown that RA-FLSs of highly inflammatory synovial tissue show a gene expression profile characteristic of myofibroblasts [23]. We detected coexpression of podoplanin and α-sma of FLSs in areas of synovial transformation and found that the expression of E-cadherin was low or absent in the podoplanin-expressing lining layer cells. We know from earlier studies that podoplanin can promote EMT of epithelial Madin-Darby canine kidney cells *in vitro *[44]. EMT is a biologic process in which polarised epithelial cells undergo sequential changes into a mesenchymal cell phenotype with increased migratory potential and the production of ECM components [24]. Loss of E-cadherin and gain of α-sma expression constitute examples of such changes. We therefore hypothesise that podoplanin is involved in an EMT-like transdifferentiation of RA-FLSs into myofibroblasts.

Podoplanin has been observed in interstitial fibroblasts in different inflammatory environments *in vivo *and *in vitro *[33,34]. In agreement with this observation, we found a locally increased expression of podoplanin in interstitial cells of the sublining connective tissue in specimens from patients with RA. However, it is difficult to determine whether upregulated podoplanin expression in the sublining in some RA specimens was a result of general inflammation or whether this phenomenon was part of a specific activation and transdifferentiation of FLSs in RA.

To confirm the specificity of the D2-40 antibody and the expression of podoplanin in RA synovial tissue, we performed SDS-PAGE and Western blot analysis of protein extracts showing a distinct band of about 45 kDa. The mature glycosylated form of podoplanin has been estimated to be about 38 to 40 kDa [27]. The difference in approximated molecular weight could be explained by the reported heterogeneity of podoplanin in SDS-PAGE, which arises as a result of heavily *O*-linked glycosylation of the core protein [27] as well as a slightly unspecific migration of the used molecular weight markers.

Characteristics of RA are the phenotypic changes and hyperplasia of FLSs of the lining layer. Conventional isolation of FLSs from synovial tissue yields homogeneous fibroblast cultures [45], but the interindividual morphological variation is large, and cultures presumably arise from both the synovial lining and sublining layers.

We established primary cultures of FLSs from human synovial tissues by enzyme digestion and found that the cells had typical fibroblast morphology. Nearly all of the primary FLSs stained positive for the fibroblast marker CD90/Thy-1 and most expressed β1 integrins. However, the IHC staining of human synovia using the anti-CD90 antibody revealed positive expression in the sublining, but not in the lining layer cells (Figures 3A and 3B). Fibroblasts possess a remarkable phenotypic plasticity [41] as well as a positional identity [46]. The synovium (lining layer versus sublining layer) of both healthy and RA patients harbour phenotypically different (by morphology and expression of surface markers) populations of fibroblasts. CD90 might therefore be a good marker for interstitial tissue fibroblasts, but not for the FLSs forming the epithelium-like lining of the synovium. In addition, fibroblasts change the expression of several surface molecules *in vitro *and acquire an "active" phenotype with prominent stress fibres and focal adhesions [47] when cultured on plastic. We therefore concluded that most of the established primary FLS cultures in this study originated from the sublining connective tissue or acquired a sublining fibroblast phenotype (with respect to CD90 expression) in culture. Using IHC, we found that cadherin-11 was expressed both in the lining layer and in cells of the sublining tissue in reactive areas, but when using flow cytometry, we found it on average in only 10% of the isolated primary FLSs. These data support the assumption that the isolated primary FLSs in these experiments originated from the sublining rather than from the lining layer.

Fibroblasts have been shown to upregulate podoplanin in culture [34]. In this study, we did not observe any significant difference in mean podoplanin expression between the RA-FLS and OA-FLS cultures. Only one culture, derived from an RA patient, was growing without contact inhibition, a characteristic of activated FLSs in RA. All cells of this culture were expressing podoplanin. Taken together, our results suggest that cultures of the lining layer FLS phenotype are hard to establish by using this technique and that primary FLSs, like other fibroblasts, probably upregulate podoplanin in culture. The observed upregulated expression of α-sma of the primary FLSs constitutes another example of an acquired feature of cultured fibroblasts.

Finally, we found a more than twofold increase in podoplanin expression in primary FLSs after stimulation with IL-1β and TNFα compared with controls. Interestingly, we also detected an increase in podoplanin expression in response to TGF-β1 stimulation. TGF-β1 is a key mediator of EMT and promotes the differentiation of fibroblasts into myofibroblasts in wound healing and fibrosis. Furthermore, TGF-β1-induced podoplanin in human fibrosarcomas [48] was found to be increased in arthritic joints in RA [49] and promoted EMT of FLS *in vitro *[13]. The fact that proinflammatory cytokines and growth factors, known to be present in high concentrations in the RA joint, stimulate podoplanin expression in primary FLSs *in vitro *supports our finding that podoplanin is upregulated in the synovium of RA patients and might be involved in the transdifferentiation of FLSs in RA.

## Conclusions

We can now add podoplanin expression as a shared characteristic of activated RA-FLSs and tumour cells that possibly affects common features of RA and carcinoma-like fibrotic tissue transformation and tissue invasion. Podoplanin might therefore be an important target not only in cancer therapy but also in the treatment of RA.

## Abbreviations

α-sma: α-smooth muscle actin; DMARDs: disease-modifying antirheumatic drugs; EMT: epithelial-mesenchymal transition; FACS: fluorescence-activated cell sorting; FLS: fibroblast-like synoviocyte; IF: immunofluorescence; IHC: immunohistochemistry; IL-1β: interleukin-1β; OA: osteoarthritis; RA: rheumatoid arthritis; TGF-β1: transforming growth factor-1β; TNF-α: tumour necrosis factor-α.

## Competing interests

MIB has received consulting fees from Schering-Plough, UCB, Pfizer, Roche and GlaxoSmithKline (less than US$10,000 each). NK was working for Bristol-Myers Squibb from 2006 to 2009 on an unrelated project. The authors declare no other competing interests.

## Authors' contributions

AKHE did the major design of the study and the coordination and establishment of the biobank, carried out the experiments, performed the imaging and FACS analysis and drafted the manuscript. TE participated in the design of the study and performed the Larsen scoring of radiographs. TE and CA included the patients in the study and collected patient data and tissue samples. CJ performed the SDS-PAGE and Western blot analyses. MB participated in the analysis of the FACS data and helped to draft the manuscript. MIB participated in the design of the study and analysis of the data and helped to draft the manuscript. All authors read and approved the final manuscript.
